# “There's More to Frail than That”: Older New Zealanders and Health Professionals Talk about Frailty

**DOI:** 10.1155/2019/2573239

**Published:** 2019-12-01

**Authors:** Susan B. Gee, Gary Cheung, Ulrich Bergler, Hamish Jamieson

**Affiliations:** ^1^University of Otago, Christchurch New Zealand, Canterbury District Health Board, Christchurch 8083, New Zealand; ^2^University of Auckland, Auckland 1023, New Zealand

## Abstract

There is general agreement that frailty is common and important in later life, but there is less agreement about what frailty is. Little is known about the extent to which practicing health professionals and older people hold a mutual understanding of frailty. Focus groups were held to engage older people and health professionals in discussion about what made them think that someone was frail. Eighteen older people took part across three focus groups, and se'venteen health professionals took part across another three focus groups. Both the health professionals and the older people talked about the experience of frailty as an interplay of physical, psychological, and social dimensions. Older people with frailty were seen as needing help and being vulnerable to adverse outcomes, but accepting help was positioned by older people as an adaptive choice. The experience of frailty was described as being mediated by the individual's psychological mindset, highlighting the importance of approaches that recognise strengths and resilience. A broader and more balanced understanding of frailty may help create more rounded and appropriate approaches to assessment and management.

## 1. Introduction

Frailty has variously been described in the medical field as “a syndrome in desperate need of description” ([1], p. 134) and in the social sciences as “one of those complex terms…with multiple and slippery meanings” ([2], p. 48). How frailty is conceptualised and understood is not merely an academic exercise: it will shape policies and access to services, care practices, and social responses and in turn the experience of frailty [3].

Within the health sector, there is a common underlying understanding of frailty as an elevated state of risk or vulnerability [4]. Older people with frailty are more vulnerable to a sudden decline in health and negative outcomes (such as hospitalisation, entry to residential care, or death) in response to seemingly small trigger events or changes—from a bout of influenza to a hip replacement [5–7]. The rates of frailty are recognised as increasing with age as a consequence of age-related physiological declines, with estimates that a quarter to a half of people aged 85 are considered frail [6]. Identifying frailty is seen as clinically useful to more effectively and appropriately target and facilitate access to care pathways, interventions, and individualised treatment plans to prevent or delay adverse outcomes [5, 8, 9].

While clinicians generally agree that they can recognise frailty and it is useful to do so, there is no consensus as to the operational definition of frailty in everyday clinical practice. One common approach is that frailty is a unidimensional medical syndrome (or phenotype) with an underlying biological cause. Frailty can therefore be measured by simple criteria-based screening tools that commonly include shrinking, weakness, exhaustion, slowness, and low activity [5, 10]. In some models, this is linked to a defining characteristic of a loss of independent capacity to carry out practical and social activities of daily living [11]. A second approach gaining popularity is that frailty represents an accumulation of a range of deficits, so that the more things a person has wrong with them, the more likely that person is to be frail. Frailty, from this perspective, is best measured using a broad index [12]. Research has found that both approaches can usefully predict negative outcomes [13–15]. There are variations and positions on a continuum between these biomedical approaches, with Hogan and colleagues cataloguing 30 different sets of criteria for what constitutes frailty. They begin their review with a quote from Lewis Carroll's Humpty Dumpty: *“When I use a word it means just what I choose it to mean—neither more nor less”* and end with a call for a framework that is relevant for both clinicians and researchers [16].

The aim of mutual understanding and usefulness can be taken a step further, to work towards an understanding of frailty that is meaningful not only to clinicians and researchers, but also to older people [17]. This meshes with a recognition that research can be a way to listen to and respect service users' experience and knowledge [18]. Compared to the extensive biomedical literature on frailty, insider perspectives on the experiences and meaning of frailty for older people are relatively scarce. Some exceptions are qualitative studies using interviews in Canada [3, 19, 20], the United Kingdom [2, 21–23], and the United States of America [2] and focus groups in Holland [17] and the United States of America [24, 25].

Within this international literature on the perspectives of older people, calls have arisen for approaches to frailty that better recognise the social and emotional expressions of frailty and reflect the lived experience of older people [2, 3, 17]. These social understandings also highlight that the label or social construction of frailty can be imposed on older people unwillingly and can contribute to negative stereotyping and the associated perceived implausibility of a good old age [2, 3, 23, 26, 27]. While there is an explicit contrast with the biomedical literature on frailty, what is less clear is the extent to which older peoples' perspectives on aging diverge or overlap with the approach of the health professionals working with this age group [28, 29].

This qualitative study explores the meaning of frailty from the perspectives of both older people and health professionals. Recognising that older people may not have preformed articulated thoughts and opinions about frailty, a focus group method was used to help participants to explore and articulate their views through group interaction [30, 31]. The aim of the present study was to explore the potential for mutual understandings amongst the perspectives of older people and health professionals to help inform clinical practice and assessment.

## 2. Methods

### 2.1. Focus Group Methodology

The primary aim of this qualitative study was to describe and understand meanings and interpretations of frailty amongst older people and health professionals, which allied with using focus group discussion to gather data. As Nyumba and colleagues assert “The most compelling reason for using focus group discussion is the need to generate discussion or debate about a research topic that requires collective views and the meanings that lie behind those views”([32], p. 28) (a., p. 28). The defining feature of the focus group is its conceptualisation as a collective conversation and the importance of the group effect [31]. The researcher acts as a facilitator, generating interaction and discussion amongst the group [31–33]. The group dynamics can “spark” clarification, reflection, justification, and exploration of the participants' own views. This allows focus group research to uncover insights and depth of discussion that may not be generated by individual interviews [31, 33–35]. It should be noted that the academic form of focus groups used in social science research has diverged markedly from the tightly structured quantitative forms of focus groups used in market research [30, 31, 35]. The academic focus group process is particularly appropriate for research that involves exploration and hypothesis building around collective views on potentially complex topics and the similarities and differences in these [33–35], as in the present study.

### 2.2. Participants

Purposive sampling was used to recruit both older people and health professional participants. A total of 18 older participants took part in the current study across three focus groups. All the older people identified as being New Zealand European. One focus group targeted younger senior citizens and was recruited through an over 65s social group and a self-improvement network. The seven participants in this group were all female, with ages ranging from 68 to 75 (average age 73.4). The remaining two focus groups targeted older-old people and were recruited from an independent living retirement village associated with an aged residential care facility. The eleven participants in these groups included three males and eight females, with ages ranging from 77 to 89 (average age 84.2). Overall, the older participants had an average age of 80.0 years.

A total of 17 health professional participants took part across three focus groups. The health professionals were recruited from the local older person's health and rehabilitation service and from relevant community organisations by a general e-mail invitation. The participants included health professionals from both inpatient and community settings. Across the three groups, there were three doctors, six nurses, four allied health professionals, one supporting role professional, and four individuals working with older people within community-based organisations. One of the health professionals identified as Māori (indigenous people of New Zealand) with the remainder being New Zealand European. There was one male health professional participant.

### 2.3. Procedure

The focus groups gathered together people who shared a key characteristic of similar age or of having a professional role serving older people, with a maximum group size of eight. Larger groups may fragment or frustrate participants waiting to have their say [32, 35]. Separate focus groups were held for older people and for health professionals to help provide an “even playing field” to facilitate comfortable collective discussion [30, 31]. A total of six groups were held, three with older people and three with health professionals. This was deemed sufficient as no new themes emerged at the third older person or health professional group [32].

The groups were held at meeting rooms at locations that were familiar to the participants. For the older people, they were held at a retirement village/aged care facility and a public library, and for the health professionals, they were held at the hospital that provides specialist older person health and rehabilitation services for the region. The discussions took place within a 90-minute session with light refreshments offered. One to two hours is a recommended length for focus groups to enable in-depth discussions without overfatiguing participants [32]. The discussions were facilitated by a researcher with experience in focus group research [33] and were audio-recorded and transcribed verbatim.

A discussion guide or “questioning route” was developed to enable consistency in the core questions used to support comparability across the groups [33]. The basic format was introduction, engagement focusing on what makes them think of a person as “frail,” exploration focusing on what makes frailty better or worse, and exit. The focus group began with a welcome and introduction to the purpose of the study and explaining the focus group process and expectations. This was followed by an “ice breaker” question answered around the group to encourage all participants to be comfortable talking and to facilitate the identification of the speakers when transcribing.

The key question in the discussion guide for the current paper was “*What's involved in being frail*?” The discussion of this topic began by the facilitator describing a scenario where they met two people they have not seen for some time, Jan and Pam. They think “Jan is getting quite frail,” but this is not the case for Pam. The participants were invited to share the “things that you think of when you think of an older person being frail.” The ensuing discussion was summarised on a whiteboard, with clustering of ideas and lines connecting clusters as interrelationships were discussed. The discussion guide suggested further prompts for the facilitator, who paraphrased and encouraged discussion, and the group participants themselves also facilitated the discussion through their interactions.

This study was approved by the University of Otago's Human Ethics Committee (reference 17/151). All participants gave informed consent.

### 2.4. Analysis

The data were analysed using the framework approach, as outlined in Table 1 [36–39]. The structure and process of the framework method of analysis offered the ability to easily compare data across the groups as well as within the groups [38]. The flexibility of the framework method enabled the researchers to begin the analysis during the focus group data collection. The data were then read and reread and then coded (conceptual/descriptive labels were applied to sections of the text) and charted. Charting is the hallmark of the framework approach and involves entering the data into a matrix with rows (cases) and columns (codes) [38]. In the present study, the cases were the individual group discussions. The final stage involved mapping, interpretation, and discussion of the themes and subthemes, with particular attention to the similarities and differences between the groups involving older people and those involving health professionals. This paper focuses on the themes related to the concept of frailty. The illustrative quotes provided use pseudonyms, and some have been abbreviated for length and clarity.

## 3. Results and Discussion

**Figure 1:**
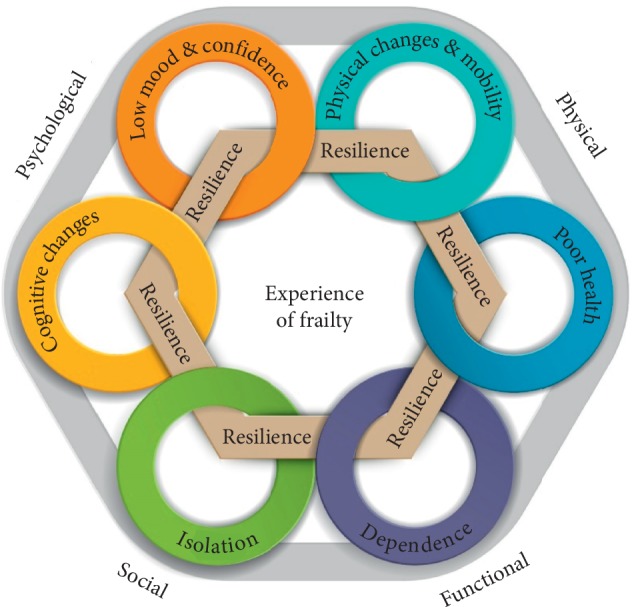
Visual summary of the thematic analysis.

This study was able to compare and contrast the way multidisciplinary health professionals and the older people talked about the frailty and found that the similarities were more pronounced than the differences in their understandings of what is involved in living with frailty. The collective perspective of the experience of frailty that emerged in the present study was developed and is represented in Figure 1. Figure 1 illustrates the breadth and complexity of considerations that may need to be taken into account to more fully understand the meaning and experience of frailty. Two overarching themes emerged.

 1: *It seems to me that frailty has quite a few dimensions*
 Frailty was discussed as a multidimensional experience encompassing challenges and losses in complexly interlinked physical, psychological, social, and functional domains:
   (i) Physical: physical changes and mobility issues and poor health
   (ii) Psychological: cognitive changes and poor mood and confidence
   (iii) Social: isolation and withdrawal
   (iv) Functional: needing help
 Each of these subthemes emerged in every group discussion. They are represented in Figure 1 by a circle of rings. An outer band represents the interplay and reciprocity of influence amongst these components.
 2: *People can have a lot of this stuff but I wouldn’t call them frail*
 The experience, impact, and acceptance of frailty were not considered to be explicable by objective losses alone. The label of frailty was contested by older people and used with caution by health professionals. Across all the groups, the experience and severity of frailty were seen as moderated by resilience and psychological resources such as a positive attitude and personality strengths.
 Resilience and psychological resources are represented in Figure 1 by an inner hexagon interlinking with the dimensions of loss. The experience of frailty in the centre of Figure 1 can be seen as being defined in concert by portions of the greater dimensions of loss and by individual resilience. While any given facet, whether sarcopenia or isolation may be part of the experience of frailty for many people with frailty, not everyone who has sarcopenia or isolation is frail.
 The visual representation in Figure 1 also provides a visual shorthand for comparing the understanding of the experience of frailty that emerged in the present study with dominant approaches to frailty. The pale blue ring representing physical changes could be considered to encompass Fried’s phenotype definition of frailty [10]. The circle of losses and challenges has similarities with summaries of the superset of deficits that might be included in a frailty index approach (e.g., [40]). While both approaches are recognisable within the narrative that emerged, neither are sufficient in themselves to represent the overall understanding of frailty that emerged in the present study. A shared understanding of frailty that balances deficits and strengths is an important foundation to integrated and person-centred care [41].

### 3.1. “It Seems to Me That Frailty Has Quite a Few Dimensions”: *Multiple Domains of Loss*

Frailty was discussed as involving challenges and losses in complexly interlinked themes across physical, psychological, social, and functional dimensions. This consistency in perspectives on what is involved in the experience of frailty was apparent despite ambiguity and differences in the definition of “frailty” per se. This more holistic biopsychosocial perspective has also emerged from interviews with Dutch elders conducted by Puts and her colleagues [17] and has been suggested in previous work with health professionals [28, 29].

#### 3.1.1. Physical Dimensions

***“Looking frail”: physical changes and mobility***

In their seminal work, Fried and colleagues identified a cluster of changes, which were then crystallised into the Cardiovascular Health Study frailty scale to become one of the most widely used frailty measures in research: unintentional weight loss, exhaustion, low energy expenditure, slow gait speed, and weakness. It is striking that this process was echoed in mental image of someone with frailty for not only the health professionals but also the older people themselves.

Observable changes around weight shrinkage and weakness were articulated across the focus groups. People with frailty were described as “*thin,*” “*drawn*,” and “*wasted*,” with “n*ot much meat on their bones*” so the “*skin just hangs*,” with the impression of getting “*smaller*” or “*shrinking*.” This appearance of shrinking can be compounded by “*poor posture*” and “*being bent over*.”

Some older people and health professionals identified “*weakness*” or decreased “*strength*” with the term “*sarcopenia*” also being used by health professionals to refer to the loss of muscle mass and strength. In two of the elder groups, this was exemplified by finding it hard to get up from the floor, as Margaret raised in the context of getting up after a fall:

  Margaret: *Well I find if I have a fall, I can't get myself up unless I crawl over to something to pull myself up.*
  Elizabeth: *Now that's the problem. That's the strength gone in your legs.*
  Judy: *We've certainly lost strength, yeah, you certainly might look alright but the strength isn't there anymore.*

There was awareness of “*low energy levels*” with the health professionals also using terms such as “*fatigue*,” “*decreased exercise tolerance*,” and “*poor energy to maintain daily living*.” Walking noticeably slower was mentioned on occasion by both older people and health professionals. A lack of physical activity was generally discussed in the context of other concepts such as a way to combat frailty or the lack of social involvement. There was however an image that people with frailty may “*just sit*” and this contrasts with those at the same age who do not have frailty, as articulated by Patrick (who is himself a member of a walking group):

*It's certainly not necessary so you only have to see [masters] games where people of a substantial age are doing all sorts of innovative and physical and social activities.*

The older people and health professionals in all the groups talked about mobility as a facet of frailty including a lack of mobility, slowness in walking, and needing aids such as a stick or walker. They noted that people with frailty may be “*unsteady*,” “*wobbly*,” or “*losing balance*.” This was associated with an increased risk of “*more*” and “*ongoing*” falls.

The role of these physical changes in determining who was frail was an area where there were divergent views. Amongst the older people, there was an explicit discussion that these features of physical changes and mobility are not in themselves enough to differentiate who is experiencing frailty:

*…but you can't do that, that's really unfair to think somebody's frail just because they've got a walker.* (Lyn, older person)

Greta later raised the opposite logical fallacy, where her mother was frail but did not look it:

  Greta: *Can I just bring up one other thing, people talking about shrinking and losing weight and things like that. Now I don't believe I'll ever look frail because I'm big and my mother was the same, I mean she lived to 91, today is the day she died, I mean seven years ago she died and, but she was big and she never looked frail, never looked frail.*
  …
  Interviewer: *So you [to Lyn] were saying it's not just about having a walker and you're saying [to Greta] it's not just about looking small…*
  Greta: *That's right, yes there's more to frail than that* (Older person group)

Patrick, a retirement village resident in another group, was very clear about making this point, for example, in the context of a fellow resident:

*I think that's perhaps being restricted, there's one particular lady in this village who uses it [a walking aid] more but, and I wouldn't describe her as frail I think she's very…alert, she's cheerful and she's accepting a physical disability but otherwise is well. So it seems to me that frailty has quite a few dimensions, it's got the physical dimension but it's also got the social and then mental … the lady I'm thinking of in the village certainly has no mental or social.*

The health professionals also talked about the physical symptoms of frailty as only being one part of the experience of the individual. For example, Claire talked about how she likes to work “*in a holistic manner*… *you're looking at the family, physical health, mental health, spiritual* …” and as the conversation continued, Kate raised the core question for considering and intervention as being “*What does that do to the whole person*?” The desire for more integrated care was expressed by both older persons and health professionals groups.

In contrast, in one of the health professional focus groups, there was the suggestion that the physical changes of Fried's phenotype were definitive of frailty. Jane, a nurse, suggested that

*…the weight loss, the lack of strength, the decreased exercise tolerance and the falls have varied degrees. And I think, like all four of those things …[are there]they are frail and then there is degrees of it…*

Although she at times struggled to articulate her conceptualisation, Jane felt that the physical changes were the core of frailty. While Jane's doubt about whether social interventions would reduce frailty was refuted by one of the other health professionals: “*I thought the evidence was very strong that it did help*,” Megan could understand the point she was trying to make

*…You can put in social stuff but they just haven't got the exercise tolerance.*

In the same group Karen also acknowledged that her biomedical background, “*my main physical side*,” biased her towards prioritising the physical changes as the core with the other dimensions as potentially preventable consequences:

*… as a [physician] I think about that [the physical side] but I would be constantly thinking, what can I do about these things to stop them impacting as much.*

Despite this definitional tension, this health professional group discussed the same multiple dimensions of the experience of frailty as the other groups.

These narratives of the commonality of the phenotype of frailty offset against its limited ability to encapsulate the experience of frailty that resonates with the argument of Cesari and colleagues: “*Although we praise this approach, our gerontological souls are still bleeding”* ([42], p. 260). The physical phenotype of frailty has proven validity but wider approaches are also needed to identify individualised targets for interventions in practice [43].

***“Poor health”: Health decline, comorbidity, and risk***

Amongst the many alternate views of frailty in the clinical and research literature, there is a common underlying core that frailty is an increased risk of vulnerability [44], that is, frailty increases the risk of poor health outcomes and creates concern for the prognosis of the individual. Comorbidity is commonly recognised as an important part of the frailty experience, as evidenced by its inclusion in over half of the most popular frailty measures [36]. The preponderance of health conditions amongst people with frailty consistently arose in the discussions across all the groups. Older people talked about “*poor health*” and being “*not well*,” along with raising specific issues such as sleep, pain, shortness of breath, continence, hearing, and vision. The health professionals also talked about comorbidities and specific conditions and additionally noted that this is often marked by multiple medications and increased hospitalisations. As with many of the dimensions, there was a vicious cycle with frailty as a risk for comorbidities and comorbidities as a risk for frailty:

*…but it was mentioned something earlier, that sort of multiple medical stuff, you just pick up. But they kind of have surgery and then something goes wrong and then they get an infection and that doesn't go and then just, so they've gone from this healthy person previously to this frail person.* (Marie, health professional)

When Greta was asked to talk more about her mother's frailty despite a robust appearance, she highlighted susceptibility to multiple health issues:

*She was very susceptible to getting things wrong with her, pneumonia…or germs yes and emphysema and yeah, and she had gout, bad gout…*(Greta, older person)

Alongside the recognition that people with frailty were susceptible to accumulating health conditions, there was also discussion that people with frailty were more likely to be slower to recover or unable to return to baseline levels of health, for example:

*Each episode takes more and more…they don't manage to recover* (Kate, health professional)

In the older persons' groups, frailty was occasionally linked to death in the discussions, for example, involving an awareness of “mortality” or causing death:

*“my personal experience of one of my parents, my dad died young, of being frail”* (Merle, older person)

The health professionals talked more extensively about people with frailty as being more “*vulnerable*” and “*closer to death* and having “*decreased reserve*” and “*less resilience*,” with even a “*minor insult causing deterioration*.” One of the health professionals used the word “*teetering*,” while Raewyn described it as being “*on the edge*”:

*…in an emergency situation, like you are looking at them and you think, they could have a fall or they could not be here tomorrow, medically, physically* (Raewyn, health professional).

#### 3.1.2. Psychological Dimensions

***“Confused and muddled”: Cognitive changes***

There have been differing opinions in the literature over the conceptual role of cognitive changes in frailty [44, 45]. There is increasing recognition of reduced cognitive reserve associated with physical frailty and its potential to improve prediction of vulnerability and outcomes such as marked declines in functioning and increased likelihood of long-term care [46–48].

The older people and health professionals in all the focus groups talked about cognitive changes with terms such as “*forgetfulness*” or memory “*failing*” or “*slipping*”; “*confusion*” or being “*muddled*,” “*vague*,” “*away with the fairies*”; “*unable to make decisions*” or “*plan*”; and cognitively “*slow*.” As with Lekan and colleagues [24], the cognitive changes described by the participants were often were broader than dementia:

*… confused and muddled. Because sometimes it's not, it's not actually confusion … …, well I was just thinking of a gentleman who I saw last week. Um, and I would have described him as frail from when, when I met him he was out pruning the roses but he looked to me, he, I would describe him as frail from the outset. The way he sort of shuffled when he walked …. And he was,…, muddled inside. You know so I was asking about his medications and he was kind of you know um, trying, almost trying to look for things and that sort of thing. And interestingly, um, there was a whole lot that I was concerned about and he passed away on Saturday morning at home.* (Kate, health professional)

*…it's a mental thing and memory is an indication of frailty and lots of people who I would class as becoming frail have a great deal of particularly memorising not only faces which we all forget but routine things like turning the stove off or switching the lights off or doing those sort of things.* (Patrick, older person)

While noting that people with dementia can be physically “*robust”* rather than frail, there was also recognition that the progression of dementia typically compromises other domains related to frailty:

*…It is very frequent that the, you know, the end stage dementia patient will be frail as well.* (Karen, health professional)

In one of the older persons' groups, Virginia, Greta, and Judy discussed some of the ways dementia can impact on other domains such as through physical changes, mobility, and social engagement:

  Virginia: *And a lot of dementia too, I've got a couple of friends with dementia which is you know, not good.*
  Greta: *And they're frail aren't they?*
 
*…*
  Virginia: *Well I s'pose it can because I watch, I watch this friend of mine and she doesn't really, I think she's forgotten how to walk properly, you know it's like she's bending, she's bending over the top, you know quite sad really.*
  Greta: *That's really sad.*
  Judy: *Well my aunt lost her voice, she's got her thoughts and she can't express herself, yeah she just, she wants to say it but she can't…*
  Virginia: *…and she's probably trying to find the words.*
  Greta: *But you were saying you had to think very hard about how you're getting up but if you've got dementia, you actually can't think about how you're going to get up…*

***“loss of confidence… with everything really”: Mood and confidence***

There was some limited discussion of being “*depressed*,” both as a feature of frailty and its role in reducing the motivation for self-care and increasing risk. This bidirectional relationship echoes the overall conclusions reached by Mezuk and colleagues following their review of the literature [49]. Based on their latent variable approach study, Lohman and colleagues suggested that psychological frailty, as measured by depressive symptoms, may be an integral part of what it means to be frail [50].

The older participants and health professionals talked more about subclinical changes, describing people with frailty as being “*introverted*,” “*withdrawn*” reduced “*vitality”* and “*engagement*,” and “*less interested in life in general*”:

  Greta: *…I sometimes feel people go quiet, I don't know why I think that*
  Jill: *They seem to take up less space don't they*

Older participants and health professionals also talked about frailty involving losing “*confidence*” and “*self-esteem*” and being “*fearful*.” For some people, this was related to things becoming harder with memory and everyday tasks so that they:

*Definitely get more nervous that they might do something wrong… lose your confidence and feeling a bit more insecure* (Brenda, older person)

For example, Judy talked about losing confidence in making decisions and worrying more and at one stage talked about this in the context of cooking:

*I used to be able to put on a dinner party for eight or nine people [or] my family. Yeah well I had three of four courses with nibbles and the whole, now I'm a mess if I've got someone coming for afternoon tea I'm trying, I have to organise it the day before and I just about lose sleep over it.* (Judy, older village resident)

This loss of confidence could be pervasive and lead to limitations in the life lived:

*… loss of confidence, um, with everything really. Could be, managing at home. Day to day living. It could be a loss of confidence in, you know leading to isolation.* (Amanda, health professional)

On occasion, older people and health professionals linked this with a loss of “self-esteem” and sense of self-identity, for example, one of the health professionals Fiona noted that people may think “I'm not sure who I am…I'm afraid to come out.”

#### 3.1.3. Social Dimension

***“Increasingly isolated”: Isolation and withdrawal***

The importance of the social dimension of the frailty experience emerged strongly from the focus groups. This reinforces previous evidence of the centrality of the social domain to the lived experience of older people. When von Faber and colleagues [51] talked with octogenarians, they found that it was the impact of limitations on social opportunities that created the greatest distress rather than the physical changes themselves. Likewise, preparatory focus groups by Studenski and colleagues as part of a process to develop a measure of frailty found that older people and their families prioritised the social and emotional aspects of frailty [52]. Social vulnerability and loneliness have reciprocal relationships with cardiovascular disease, depression, and dementia [53] and independently predict high rates of entry into aged residential care [54] and mortality [55].

Older people with frailty were described as “*lonely*” and *“isolated*,” lacking “*social networks,”* “*social connections,”* and *“social stimulation.”* There was description of a “*narrowing in the world*,” or what Studenski and colleagues [52] call the life space and social world. There was considerable discussion amongst the older participants about the complex interplays between the different facets of frailty and social difficulties whether physical features such as loss of mobility, hearing, sight, or continence; psychological features such as cognitive changes, confidence, and social withdrawal; or environmental factors such as lack of transport or social opportunities where they live or the loss of an enabling partner or roles. Hearing was often a particularly salient example to the older people. For example:

*I'm wondering about people who, in their life have been social and yet now because they're deaf and they can't see and they can't drive and you know, and they've got lonely because in the past they've gone out to volunteer wherever and suddenly they can't so yeah……there's something more where they haven't got the choice because there's something that happens* (Jill, older person)

In another example, one of the older groups talked about how some older people do not take up social opportunities:

  Elaine: *Well they could have other problems, like hearing problems, sight, problems, that if they can't hear they don't enjoy…*
  Judy: *No that's right, or it's just a jumble and my husband used to have a hearing aid and said it was terrible, he used to turn them off …*
  Prue: *There's certain things in your life too that when you lose your partner…it's very hard to make that effort to come on your own when you've always been together…You just feel quite lost, some people go into themselves and withdraw*

There was also talk amongst the health professionals of how social engagement is impacted by multiple factors. For example, Shona noted the interplay amongst sensory issues, mobility, continence, self-esteem, cognitive skills, and socialisation and summed up:

*…it's really [being] vulnerable isn't it? You're kind of saying that you know if you have problems with any of these [factors], it becomes so much harder to get out and socialise…* (Shona, health professional)

For Claire, social isolation was the apex of the frailty experience:

*Social stimulation. I think is such a big one. Like um, whether it's, you know if they're at home and they have difficulty getting out but if they're still being visited… But I would say isolation is, is almost the, the thing that breaks the camel's back almost. Yeah. That, you know a lot of these other things will affect their ability to get out of the house or you know they might have lost their drivers licence or whatever, and then increasingly isolated. Just has such a big impact on people.* (Claire, health professional)

The importance of the social dimension of frailty in this and previous work suggests that not addressing this dimension may run the risk of misalignment with what matters most to some older individuals. For example, a recent publication on assessment of frail older people in acute settings recommends an assessment package encompassing a physical exam, psychiatric exam, functional assessment, and a history of gait and falls, continence, sensory problems, and medications—noticeably absent from this summary of recommendations is assessment of the social dimension [56].

#### 3.1.4. Functional Dimension

***“Need help”: Dependence***

Both the community participants and health professionals talked about people with frailty not being able to do the same things independently, particularly day-to-day tasks, with the common understanding that part of frailty is needing more “*support*” and “*help*” from family and health services, or more pejoratively being more of a “*burden*”:

*It gets to the stage where you really do need help”* (Margaret, older village group)

*I think it's probably when they start to lose the, yeah the processing and the ability to manage independently …More of an effort. Because they could be all that and they'd be fine…But it's when they start not being able to manage that independence.* (Megan, health professional)

The need for help as a marker of frailty arose in every focus group, echoing the emphasis that Rockwood and colleagues have placed on dependence as the crux of clinical judgement about frailty [57]. As Landi et al. [45] note, the ability to maintain independent has multiple determinants and is sensitive to changes in cognition, mood, mobility, and functional performance.

Accepting help can key into pervasive narratives that needing help is a marker of adverse outcomes or unsuccessful aging in Western cultures that prioritise independence:

*sometimes that, I would think are reluctant to accept support is that, ah well if they accept support then they've given up* (Claire, health professional)

There was resistance to this narrative amongst the older participants. Butler and colleagues have argued [58] accepting help can be an adaptation to improve quality of life, rather than resisting help and risking exacerbating problems. They have labelled this adaptation “responsible dependency.” Accepting help is a choice:

*I'm thinking all these things, everything …the people concerned have to make the decision to use all the facilities, all the help…* (Beth, older person)

Judy was aware that her own habit of declining help to maintain independence and not be a burden could mean turning down help that would have had a positive impact:

*I think it's our own fault ‘cause we've always been so independent…we've never asked them for help… if they say you know, oh can we, oh no no we're alright, yeah and that, and [my husband] and I, that's what we decided, we'd come in here, we wouldn't be a drain on them and that's what I think is a bit of a trouble…*

Some of the women felt that men found it particularly difficult to seek help, with concomitant risks:

*I think they're more fearful about admitting and more fearful about what might happen to them. My husband died at 68 and he was obviously having heart problems but didn't say so and dropped down dead on the pavement so had he sought medical help, whether it was for fragility or anything else, he'd still be here today.* (Lyn older person)

William explained the aspect of responsibility clearly in relation to using mobility aids:

*I think one has to accept as one gets older that one is going to have certain problems and if you can take precautions so that you don't injure yourself or others, you take those precautions, in other words, you use a stick or an aid of some sort.*

### 3.2. “People Can Have a Lot of This Stuff but I Wouldn't Call Them Frail”: Resistance, Resilience, and Psychological Resources

Frailty index models view frailty as a multifactorial accumulation of deficits that places the person at risk of adverse outcomes, and offer an approach which can incorporate physical, psychological, and social dimensions (e.g., [7]) (although not all authors incorporate the full range). While the emphasis on the prediction of adverse outcomes by cataloguing objective limitations can be useful, arguably this approach in isolation is not geared to understand and reflect the holistic lived experience and meaning of frailty for the individual [11, 19]. Lived experience is more than a problem list [27].

***“I don't use the word frail”: Resistance to the label of frailty***

Authors have explored how the focus on defining frailty by deficits has unwittingly positioned frailty as the repository for negative stereotypes and visions of a feared and unsuccessful old age—what once would have been termed senescence or infirmity [26, 27]. This echoes the distinction between a healthy third age and a fourth age marked by loss of capacity, with frailty as the boundary [26]. Little wonder then perhaps that as with previous studies [2, 17, 19, 23] we found resistance to the label of frailty. The older people made comments that rejected the label as applying to them: “*We don't think we're frail anyway so*” (Elizabeth); and that frailty was not a word that they would use; “*I've gotta say I don't use the word frail*” (Keith). Christine, in particular, questioned the imposition of frailty as a label: “*who has the right to say you're frail in any way?*,” while William railed against his health concerns being written off: “*Yeah I'd like people not to use the phrase…What do you expect at your age*.”

The health professionals were aware of these tensions and cautious about using the term. Some of the health professionals would not tell an individual that they were frail or only if they were sure of the relationship. Some professionals noted that they had been involved in discussions about avoiding using the term frailty in interdisciplinary team meetings or in materials. Raewyn was aware that the frailty label was affixed to older people by health professionals, sometimes inaccurately in her opinion, and that this could have implications for clinical decision making and the older person's hope for a more positive future:

*… a lot of that is happening where people are being given that label and um, is that impacting on what health care they have been given as well. You know, um, oh she is old and frail and we see that a lot, actually, oh they are frail.…Um, some of them laugh it off, oh he doesn't know what he is talking about or whatever. But um, no it does have an impact because they almost give up on some things. Oh, we can't, I am not going to bother to push for that um show the surgery or whatever or you know, getting that fixed up because I am told that um, I am old and frail.*

***“They are still cheerful and they are still resilient”: feeling frail vs. resilience***

Markle-Reid and Brown [11] have argued that any understanding of frailty must recognise that the degree of frailty depends in part on the context and can be highly influenced by the subjective interpretations of the individual. This was dramatically highlighted in Merle's story about her mother:

*Well my mum lived till nearly 101……[at 96] she was still not frail, she was robust…but at 96… the doctor said she had bowel cancer, well she just took to the bed, I mean we were absolutely amazed and “you girls can do what you like with me,” she became old…rapidly and then I went to see her next time, she'd lost a lot of weight and had a walking stick so you know, within no time at all. However once she discovered she didn't have bowel cancer…She immediately felt better…so then she leapt out of bed and got on with her life again…*

The importance of this subjective context-dependent perception was a key theme emerging from Grenier's interviews with older women, which she called “feeling frail” [19]. Grenier [19] noted from her qualitative work that moments of feeling frail do not always result in a final state of being frail. This is illustrated by Virginia in the present study who shared her own experience of “feeling frail” after an illness requiring hospitalisation:

*I just recently got quite ill and I felt very frail, my children were wanting to put me in a you know, retirement [home]… and I've now come right so I'm feeling much but I was staying at home and feeling quite old…and yet I'm very active and healthy and outgoing and can speak my mind and all the rest of it but I just felt, oh someone come and look after me, please…but I didn't [call it] being frail but now that we're talking about it, that was actually how I felt at the time, I don't anymore but…Yes it can be a bit of a mindset too, a bit in your mind I felt afterwards…*

From her work, Grenier [19] has suggested that there is an “emotional threshold” for frailty and not just a medical-functional threshold. One of the health professionals puts it this way:

*there's a whole lot of these things that can happen, people can have a lot of this stuff but I wouldn't call them frail, not necessarily because it's depends on how they see life* (Marie, health professional)

Feeling frail is not just a consequence of a change in health and functioning but also a determinant [23]. This allows recognition of the role of emotional resilience in understanding individual differences in outcomes in the face of major challenges, as in this discussion:

  Jane: *It's a funny thing, I mean you look at the level of function they have and they are still cheerful and they are still resilient and independent and they are making do …they look like they would blow over in the next wind but still somehow pushing in through*
  Raewyn: *So, strong in mind would jump into that category…*
  Karen: *and some have really one thing wrong with them and they give up and allow themselves to fall into the sick role and very quickly become frail because that is how they believe, that is their role in life* (health professionals group)

Participants talked about being “*a strong person*” and the impact of “*personality*.” The concept of resilience can be described as a personal characteristic that enables an individual to sustain, regain, or achieve physical or emotional health in the face of illness or loss [59, 60]. However, still a developing research area, a resilience-based approach may help to counteract the potential for negativity and stigmatization [61] and mesh with a strength-based approach to services for older people. The foundation of a strength-based approach is that each person has abilities and resources that can help them to cope with challenges. Dury and her colleagues have talked with older frail people about the multiple positive balancing factors that the experience of frailty, including from the individual themselves [62]. They can use their experience and capabilities to identify their own concerns and be involved in the process of regaining, maintaining, or adapting to their level of health [63]. A person-centred strength-based approach places the unique strengths and preferences of the client in the context of their lived environment into the central focus of the helping process, rather than the client's problems, diagnoses, or deficits [64].

## 4. Limitations

The present study adds a New Zealand perspective to the growing literature base that both older people and health professionals commonly view the experience of frailty as more than just a physical change. While the study involved a range of health professionals, there are discipline and speciality differences that remain unexplored in this study. The groups of health professionals each included a range of disciplines reflecting the multidisciplinary nature of their older persons' health work environments. Their multidisciplinary experiences in this field may have made it more likely that they held more holistic views of the experience of ageing than health professionals who work with the general population. It would be particularly useful for future research to explore the perspectives of general practitioners. The older participants were limited to individuals from the majority New Zealand European population and to those who felt comfortable with coming to a group to talk about frailty and were able to attend. The groups may not reflect the views of the most vulnerable older people such as those who have severe frailty or isolation. Comparing and contrasting the perspectives of older people with frailty with those who are robust may be a possible area for future research. Future studies with ethnic minority groups would be useful, as would studies from non-Western countries. For a better understanding of the experience and meaning of frailty to impact on the well-being of older people, that understanding must be reflected in practice. How to effectively structure, encourage, and evaluate communication and needs assessment processes about frailty that are holistic and strength-based will be an important area for future research.

## 5. Conclusions

The medical and social perspectives on frailty have tended to be in separate siloes; however, integrating these approaches may provide an impetus to strengthen person-centred services [59]. As Nicholson and colleagues remind us, a shared understanding of frailty between older people and health professionals is a good basis for shared decision making [64]. The findings of the present study support calls for a more holistic approach to the assessment of the needs of frail older people that includes psychological and social domains [24]. This study was distinctive asking the same questions of older people and health professionals, and the discussion suggests that embedding a more holistic approach will support the broader understandings of the experience of frailty already held by many of the health professionals who work with older people [28, 29]. This is exemplified in the British Geriatrics Society's “Fit for Frailty” best practice guidelines [65]. These guidelines recommend conducting a holistic and comprehensive review of needs, not just medical but also functional, psychological, and social, to inform a personalised care and support plan which incorporates the individual's goals. The older people and health professionals in this study strongly identified the role of psychological resilience and recognised that the degree and impact of frailty, and the support that is appropriate, is in part dependent on the individual's subjective perceptions and other balancing factors [61]. A recognition of the strengths and resources of the individual is a central tenant of a person-centred approach. A more balanced approach to understanding the individual's strengths as well as limitations may help transform perceptions that frailty is a nihilistic all-encompassing label. Regardless of the specifics of the mechanisms of the interrelationships amongst the domains of the frailty experience, recognising the intrinsic connections between the biopsychosocial aspects of the frailty experience will improve our understanding, predictions, and decision making [50]. A broader shared understanding of frailty and the strengths of the older individuals experiencing it can help us to place focus on the interventions that matter most to individual and help to create a more positive and rounded experience for those we seek to help [11, 59].

## Figures and Tables

**Table 1:** Steps in the framework analysis used in the present study.

Stage	Process
1. Familiarisation	Through the process of note-taking in the focus groups and reading and rereading the transcripts of the discussion and the whiteboard summaries, the researchers became aware of the recurring themes and key ideas. Initial thematic notes were made and discussed.

2. Identifying a thematic framework	The key ideas and themes that were identified in the familiarisation stage formed the basis for an initial thematic framework that was used to classify the data. Open coding ensured that any important themes from the data that had not been captured initially were able to be included and the framework was adapted as necessary.

3. Indexing	The transcripts were annotated to identify sections that were relevant to the different codes or labels.

4. Charting	An excel spreadsheet was used to generate a matrix, with the groups as rows and the labels as columns. Quotations identified in the indexing were entered into the matrix in the appropriate cell using verbatim words.

5. Mapping and interpretation	The matrix was reviewed with reference to the transcripts and team discussion to clarify the main themes and subthemes and the interrelationship between these. The matrix structure enabled easy recognition of patterns, in particular whether there was consistency of a theme across the groups versus some empty cells.

## Data Availability

The anonymised transcripts used to support the findings of this study are available from the corresponding author upon request.
